# Complex Cesarean Myomectomy: A Case Report Describing the Removal of 15 Fibroids During Delivery

**DOI:** 10.7759/cureus.78637

**Published:** 2025-02-06

**Authors:** Divya Mishra, Shantanu Shubham

**Affiliations:** 1 Obstetrics and Gynaecology, Graphic Era Institute of Medical Sciences, Dehradun, IND; 2 Neonatology, Graphic Era Institute of Medical Sciences, Dehradun, IND

**Keywords:** cesarean myomectomy, hemorrhage, large fibroid, multiple fibroids, pregnancy

## Abstract

Cesarean myomectomy (CM) has traditionally been avoided due to concerns about hemorrhage and the potential need for peripartum hysterectomy, yet recent evidence suggests it can be a safe and effective procedure in selected cases. This report details a rare and complex CM performed on a 40-year-old primigravida with a history of fibroid uterus and infertility. The patient presented with multiple uterine fibroids, including a 15 cm intramural fibroid in the lower uterine segment obstructing the proposed cesarean incision. At 36 weeks, a planned CM was performed, excising 15 fibroids (2-15 cm). Hemostasis was achieved and blood loss was limited to 700 mL. The procedure lasted two hours and 10 minutes. Both mother and neonate recovered well and were discharged on day 4 without complications and have been healthy as of the six-month follow-up. This case highlights the feasibility of CM in managing large and multiple fibroids, emphasizing the importance of experienced surgical expertise and advanced hemostatic techniques.

## Introduction

Uterine myomas, or fibroids, are noncancerous tumors frequently observed in women, with a prevalence of approximately 40-50% by the age of 35 and rising to around 70-80% by the age of 50 [1]. During pregnancy, the occurrence of myomas ranges between 3% and 10%. The likelihood of developing myomas increases with age, with the highest prevalence typically seen during the fourth decade of life [2]. They can complicate pregnancy, increasing the risk of preterm labor, miscarriage, and obstructed delivery. Cesarean myomectomy (CM), the surgical removal of fibroids during cesarean section, has historically been approached with caution due to concerns of significant hemorrhage and the potential need for hysterectomy [3]. However, advancements in surgical techniques and a better understanding of the procedure's risks and benefits have led to its increased acceptance in carefully selected cases [4].

This report describes a landmark case involving the removal of 15 fibroids, including a 15 cm intramural fibroid obstructing the proposed incision site, during a cesarean section at 36 weeks of gestation. The procedure demonstrates that CM, though not universally standardized, can be a feasible option in high-risk pregnancies. It highlights the importance of individualized care and skilled surgical expertise in achieving favorable outcomes in challenging cases, offering new possibilities for managing uterine fibroids during pregnancy.

## Case presentation

A forty-year-old primigravida presented at 30 weeks of gestation with a four-year history of fibroid uterus. She was a known case of hypothyroidism and had no other significant comorbidities. Her ultrasound (USG), six months before conception had revealed the size of the largest fibroid to be 4 cm. She had regular antenatal visits, and initial USG during her first trimester revealed four fibroids, the largest measuring 7 cm. By 32 weeks, this had grown to 15 cm, significantly distending the uterus. The antenatal course was uneventful except for persistent heaviness in the lower abdomen after 20 weeks of gestation along with occasional episodes of pain abdomen requiring the use of analgesics. There was no growth abnormality in the fetus. The baby was appropriate for gestational age and did not have any deformity. The patient was counseled for MRI prior to delivery but due to financial constraints, it could not be done. She was admitted at 32 weeks in threatened preterm labor during which antenatal steroid coverage was also done. Between 34 and 35 weeks, she had three visits to the emergency with complaints of pain in her lower abdomen, though not in labor. Urine examination was normal during these visits and the pain was attributed to the overdistension of the uterus. Considering the constant discomfort of the patient and anticipating the risk of fibroid-related obstruction and preterm labor decision was taken to conduct delivery at 36 weeks.

Intraoperative findings and procedure

**Figure 1:**
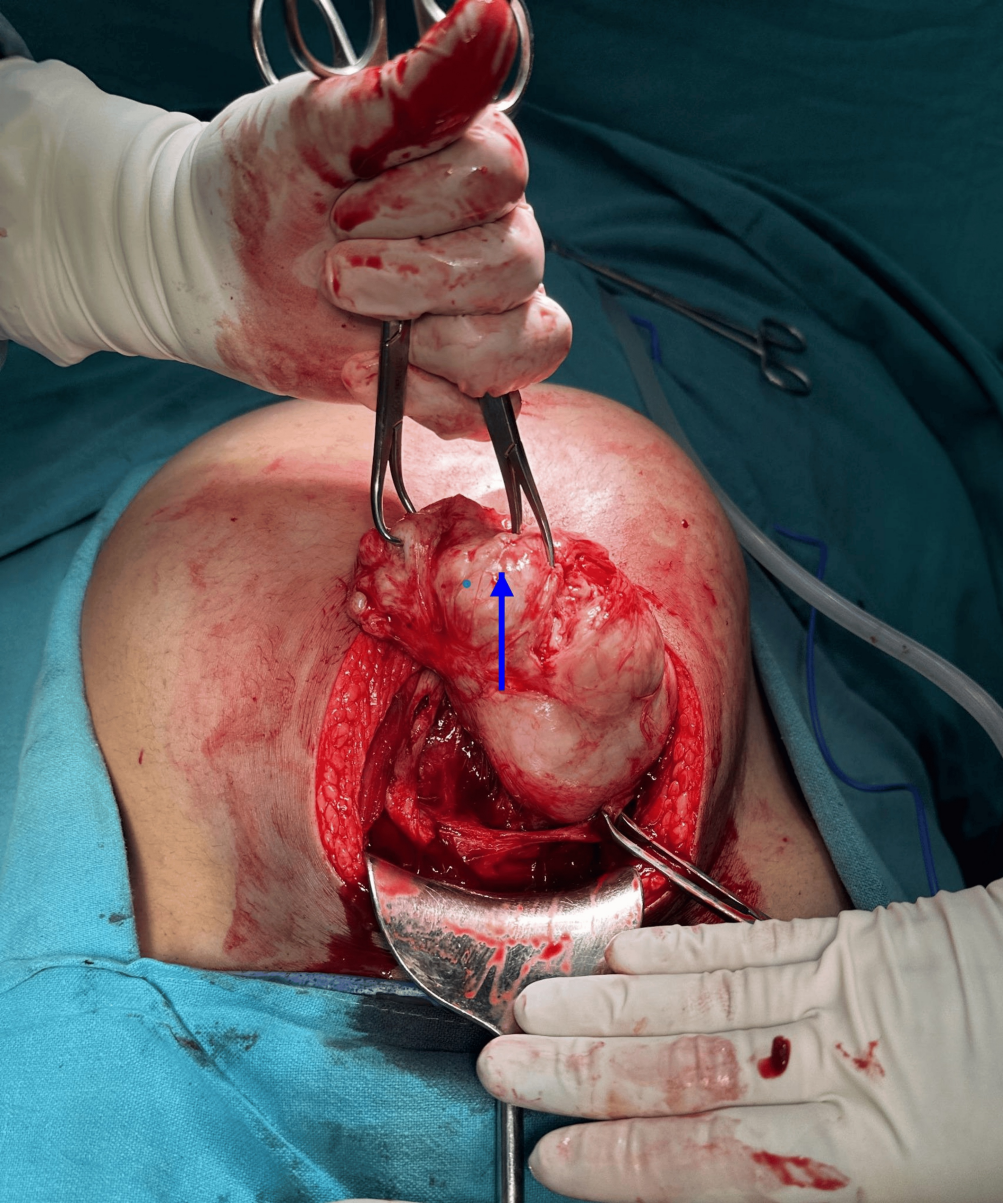
A large 15 cm fibroid obscuring the LSCS incision site LSCS: lower segment cesarean section

The surgery was done under spinal anesthesia. Upon entering the abdomen, the largest fibroid was identified intramurally in the anterior lower uterine segment, obstructing the planned lower segment cesarean section (LSCS) incision. Though the procedure was originally a planned CM only, performing only LSCS without myomectomy was no longer an option as the largest fibroid was completely obscuring as well as distending the lower uterine segment and more than half of the adjoining upper uterine segment. Delivering the baby without removing the fibroid was practically not possible. A decision was made to proceed with the enucleation of the large fibroid to create adequate access for the surgical incision required to safely deliver the baby (Figure 1).

The abdomen was opened by a midline vertical incision. On the uterus, a lower segment transverse incision was given, which was overlying the prominent part of the fibroid. After the removal of the fibroid, the rest of the uterine layers were opened along the same incision to deliver the baby. The enucleation allowed for the restoration of uterine anatomy, enabling a controlled and safe delivery. After enucleating the first myoma which was lying over the planned LSCS site, a full-thickness incision was given over the uterus including the area underlying the myoma bed. The baby was in cephalic presentation and cried immediately after birth; delayed cord clamping was done and the baby did not require neonatal intensive care unit (NICU) admission. The APGAR scores were 8 and 9 at one and five minutes, respectively. There was no excessive bleeding and the myoma was enucleated smoothly without any other complications. The placenta along with membranes was removed completely by controlled cord traction.

**Figure 2:**
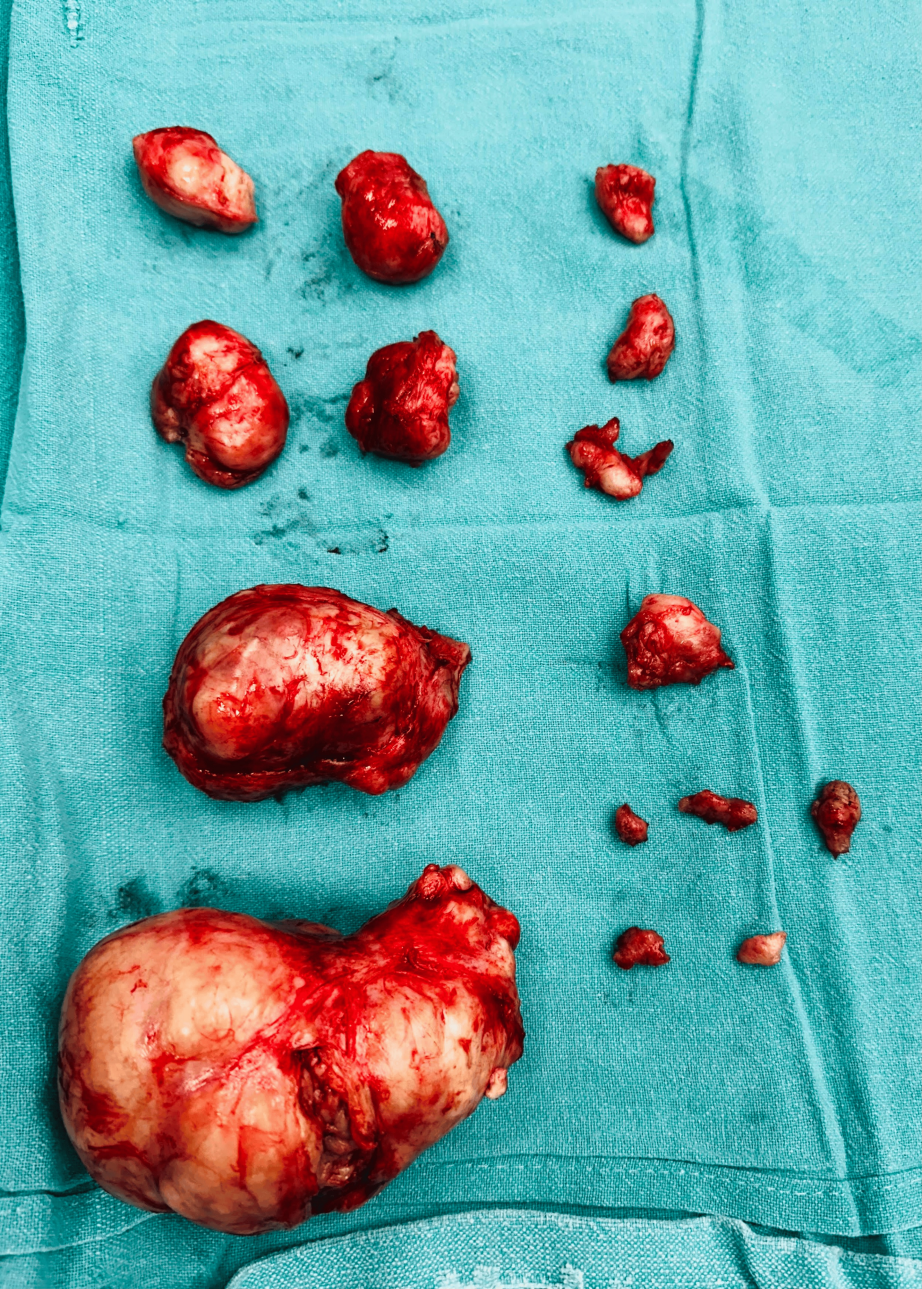
A total of 15 fibroids removed during surgery, size ranging from 2 cm to 15 cm in greatest dimension

We initially anticipated the presence of four fibroids based on prior imaging. However, intraoperatively, the uterus was found to be studded with numerous myomas of varying sizes, predominantly intramural, located on both the anterior and posterior uterine walls and fundus. These fibroids ranged from 2 cm to 15 cm in their largest dimension, with five fibroids exceeding 5 cm. In order to minimize blood loss, vasopressin was injected into uterine musculature. Twenty units of vasopressin were diluted in 200 ml of normal saline and 120 ml of this solution in total was used, after which the uterus was blanched sufficiently. Additional fibroids were identified and meticulously excised, totaling 15 in number (Figure 2).

The specimen was sent for histopathology and was reported as leiomyoma. A barbed suture was selected in order to prevent excessive blood loss and the formation of hematoma in the myoma bed later on. As a hemostatic measure, we used injection vasopressin in addition to barbed suture. The use of a tourniquet was not feasible because of the shape and location of myomas. The abdomen was opened by a midline vertical incision to ensure adequate accessibility, the myoma bed was sutured in layers, and the final closure of myoma defects was done using the baseball suture technique. The cesarean incision was subsequently closed after ensuring adequate hemostasis and uterine integrity. The procedure was completed in two hours and 10 minutes, with an estimated blood loss of 700 ml. The patient endured the procedure well and remained hemodynamically stable throughout the surgery.

Postoperative course

The mother was monitored in the recovery ward following surgery and remained stable throughout the observation period. Four hours after the procedure, she was transferred to the postnatal ward as her vitals were stable, with no signs of postpartum hemorrhage.

A soft diet was initiated eight hours after surgery, and limb mobilization was encouraged. Full ambulation out of bed was achieved 24 hours postoperatively, along with the removal of the urinary catheter. Her recovery was smooth and uneventful, comparable to that of a standard LSCS patient. Notably, she did not require any blood transfusion preoperatively, intraoperatively, or postoperatively. Her preoperative hemoglobin was 12.2 g/dL, and postoperative hemoglobin was 10.1 g/dL. The neonate was shifted with the mother, was exclusively breastfed, and showed no complications. Both the mother and baby were discharged on postoperative day 4 in stable condition. The mother is under regular follow-up care and is healthy and complication-free up to the most recent follow-up at six months post-surgery. A scan was done three months after the surgery, which showed a normal uterus with two sub-centimetric intramural fibroids.

## Discussion

CM remains a complex and challenging procedure due to the inherent risks, including significant hemorrhage, peripartum hysterectomy, blood transfusion, intensive care requirements, prolonged hospital stay, and associated morbidities. However, this case emphasizes that with meticulous preoperative planning, judicious intraoperative measures, and skilled surgical execution, CM can be performed safely, even in highly complex scenarios [5,6].

Case reports have highlighted successful outcomes in CM involving large fibroids. For example, one report described the removal of large fibroids (>15 cm) during CM, with patients experiencing a decrease in hemoglobin but not requiring blood transfusions. The patients were discharged in good condition and had no chronic pelvic pain or menstrual abnormalities post-surgery [7]. Another case involved the removal of eight fibroids during a cesarean section without maternal or fetal complications, highlighting the potential for successful outcomes even with multiple fibroids [8].

Notably, there are instances where avoiding myomectomy during a cesarean section is no longer a viable option. In this case, the presence of a large fibroid completely obstructing the planned incision site made myomectomy indispensable to enable safe delivery of the baby [9]. Such situations highlight the importance of surgical preparedness and adaptability, as imaging may not always accurately predict the number, size, or location of fibroids encountered intraoperatively.

Several studies have demonstrated that CM can be performed safely without increasing maternal morbidity. For instance, a retrospective cohort study found no significant difference in blood transfusion requirements or postoperative hemoglobin levels between women who underwent myomectomy during cesarean section and those who did not, although the operative time and hospital stay were longer for the myomectomy group [10].

Another study highlights the safety and feasibility of performing CM in a tertiary care setting. It compared outcomes between 83 patients undergoing CM and 80 undergoing cesarean section alone. Despite increased surgical time and hospital stay for CM, no significant differences were observed in complications or blood transfusion rates, suggesting that CM, when performed by experienced surgeons with proper planning, is a safe option for selected cases [11].

The practice of performing myomectomy during a cesarean section remains a controversial subject in contemporary obstetrics. Historically, this procedure was largely avoided due to perceived risks, except in cases involving pedunculated subserosal fibroids. However, recent studies have demonstrated that, when conducted by skilled obstetricians, the surgery is safe and does not result in significant complications [12]. A retrospective case-control study evaluated 40 women with fibroids who underwent CM and compared them with 80 women with fibroids who had cesarean sections without myomectomy. The results revealed no notable difference in hemorrhage rates between the two groups, which were 12.5% and 11.3%, respectively [13]. Another study involving 12 patients who underwent CM observed that enucleation was significantly easier during pregnancy, attributed to the increased tissue softness during this period [14].

It is imperative to develop and refine the expertise required for CM, both in planned and unavoidable emergency scenarios. This skill set is crucial for preserving the uterus post-procedure and minimizing delays in delivering the baby due to indecision during surgery. Mastery of these techniques contributes significantly to improving maternal and neonatal outcomes, ensuring the safety and feasibility of CM in challenging obstetric cases.

## Conclusions

This case highlights the feasibility and safety of CM in managing large and multiple fibroids. Despite traditional concerns regarding hemorrhage and peripartum hysterectomy, it demonstrates that with meticulous preoperative planning, advanced hemostatic techniques, and skilled surgical expertise, successful outcomes are achievable. The removal of 15 fibroids, including a 15 cm intramural fibroid, during a CM emphasizes the adaptability required in obstetric surgery. Both mother and neonate recovered smoothly, reaffirming that CM, with individualized care and surgical preparedness, can improve outcomes in select high-risk pregnancies.

## References

[1] Johnson NL, Norwitz E, Segars JH (2013). Management of fibroids in pregnancy. Fibroids.

[2] Ciavattini A, Clemente N, Delli Carpini G, Di Giuseppe J, Giannubilo SR, Tranquilli AL (2015). Number and size of uterine fibroids and obstetric outcomes. J Matern Fetal Neonatal Med.

[3] Song D, Zhang W, Chames MC, Guo J (2013). Myomectomy during cesarean delivery. Int J Gynaecol Obstet.

[4] Roman AS, Tabsh KM (2004). Myomectomy at time of cesarean delivery: a retrospective cohort study. BMC Pregnancy Childbirth.

[5] Huang Y, Ming X, Li Z (2022). Feasibility and safety of performing cesarean myomectomy: a systematic review and meta-analysis. J Matern Fetal Neonatal Med.

[6] Zemet R, Dulitzki M, Baum M, Ofer Friedman H, Morag I, Simchen MJ (2021). Early-onset preeclampsia - the impact of antiphospholipid antibodies on disease severity. Eur J Obstet Gynecol Reprod Biol.

[7] Tjokroprawiro BA, Saraswati W, Yuliati I (2021). Successful cesarean myomectomies of large uterine fibroids: two cases and a literature review. Am J Case Rep.

[8] Meirow D, Schenker JG (1995). Cancer and male infertility. Hum Reprod.

[9] Garg P, Bansal R (2021). Cesarean myomectomy: a case report and review of the literature. J Med Case Rep.

[10] El-Refaie W, Hassan M, Abdelhafez MS (2020). Myomectomy during cesarean section: a retrospective cohort study. J Gynecol Obstet Hum Reprod.

[11] Sakinci M, Turan G, Sanhal CY (2022). Analysis of myomectomy during cesarean section: a tertiary center experience. J Invest Surg.

[12] Guler AE, Guler ZÇD, Kinci MF, Mungan MT (2020). Myomectomy during cesarean section: why do we abstain from?. J Obstet Gynaecol India.

[13] Fukui O, Shimoya K, Shimizu T, Fukuda H, Wasada K, Murata Y (2005). Helicobacter pylori infection and platelet counts during pregnancy. Int J Gynaecol Obstet.

[14] Kwawukume EY (2002). Myomectomy during cesarean section. Int J Gynaecol Obstet.

